# A Multiparametric MRI-based Radiomics Model for Stratifying Postoperative Recurrence in Luminal B Breast Cancer

**DOI:** 10.1007/s10278-023-00923-9

**Published:** 2024-02-29

**Authors:** Kepei Xu, Meiqi Hua, Ting Mai, Xiaojing Ren, Xiaozheng Fang, Chunjie Wang, Min Ge, Hua Qian, Maosheng Xu, Ruixin Zhang

**Affiliations:** 1 Department of Radiology, The First Affiliated Hospital of Zhejiang Chinese Medical University (Zhejiang Hospital of Traditional Chinese Medicine), Zhejiang Province, Hangzhou, China; 2 The First School of Clinical Medicine, Zhejiang Chinese Medical University, Zhejiang Province, Hangzhou, China; 3 Department of Radiology, Hangzhou First People’s Hospital, Zhejiang Province, Hangzhou, China

**Keywords:** Radiomics, Magnetic resonance imaging, Recurrence, Luminal B, Breast cancer

## Abstract

This study aims to develop an MRI-based radiomics model to assess the likelihood of recurrence in luminal B breast cancer. The study analyzed medical images and clinical data from 244 patients with luminal B breast cancer. Of 244 patients, 35 had experienced recurrence and 209 had not. The patients were randomly divided into the training set (51.5 ± 12.5 years old; *n* = 171) and the test set (51.7 ± 11.3 years old; *n* = 73) in a ratio of 7:3. The study employed univariate and multivariate Cox regression along with the least absolute shrinkage and selection operator (LASSO) regression methods to select radiomics features and calculate a risk score. A combined model was constructed by integrating the risk score with the clinical and pathological characteristics. The study identified two radiomics features (GLSZM and GLRLM) from DCE-MRI that were used to calculate a risk score. The AUCs were 0.860 and 0.868 in the training set and 0.816 and 0.714 in the testing set for 3- and 5-year recurrence risk, respectively. The combined model incorporating the risk score, pN, and endocrine therapy showed improved predictive power, with AUCs of 0.857 and 0.912 in the training set and 0.943 and 0.945 in the testing set for 3- and 5-year recurrence risk, respectively. The calibration curve of the combined model showed good consistency between predicted and measured values. Our study developed an MRI-based radiomics model that integrates clinical and radiomics features to assess the likelihood of recurrence in luminal B breast cancer. The model shows promise for improving clinical risk stratification and treatment decision-making.

**Supplementary Information:**

The online version contains supplementary material available at 10.1007/s10278-023-00923-9.

## Introduction

Breast cancer is the most common malignancy worldwide and a leading cause of cancer-related deaths in women [1]. The recurrence of breast cancer often indicates a poor prognosis [2], with hormone receptor-positive breast cancer being the most common subtype. Luminal B breast cancer, a more aggressive subtype, has a higher risk of recurrence than luminal A breast cancer due to its higher proliferative capacity and lower progesterone receptor (PR) expression [3, 4]. While additional treatment is often administered to luminal B patients to reduce the risk of postoperative recurrence [5], proper stratification of recurrence risk is essential for guiding treatment decisions as patients with low recurrence risk may become over-treated [6, 7].

Currently, traditional histopathological factors such as tumor size, lymph node status, and histology grade [8, 9] are used to make adjuvant therapy decisions. However, these methods may not fully characterize the complexity of the molecular biology of tumors, and patients with similar characteristics may have different clinical outcomes. The 21-gene recurrence score (RS) testing provides prognostic information that is independent of clinicopathological features [10], but it is an expensive and invasive method. Therefore, a non-invasive and comprehensive method that allows the assessment of tumor heterogeneity is needed to assist in individualized patient treatment decisions.

MRI is a valuable tool for the diagnosis, evaluation, and prognostic evaluation of breast cancer. Not only does it enable dynamic and holistic assessments of tumors, but it also provides non-invasive characteristics of tumor morphology and function [11]. Radiomics is a technique that analyzes high-throughput image features automatically, which enhances the characterization of images by detecting features that cannot be detected by human eyes [12]. Radiomics models based on MRI images have shown promise in stratifying the recurrence of hormone receptor-positive breast cancer [13]. Few studies have specifically examined luminal B breast cancers, despite the potential for models based on a single molecular subtype to identify the intrinsic characteristics of subtypes and improve their accuracy. In previous studies, Yang et al. further subdivided the patients with luminal B breast cancer using PR and Ki-67 index and found that the new classification performed better in predicting the survival outcome and recurrence score [14], whereas Xiong et al. discovered that the ultrasonography-based radscore could be used to differentiate between patients at high and low risk of recurrence in luminal B breast cancer [15]. However, neither of these two studies used features related to MRI radiomics or combined multiple features, such as clinical features, which may limit the ability to stratify recurrence in luminal B breast cancer. Therefore, our study aimed to evaluate whether a multiparametric MRI radiomics model combined with clinicopathological characteristics can be used to assess and stratify the postoperative recurrence risk of patients with luminal B breast cancer.

## Materials and Methods

### Patient Cohort and Data Collection

**Fig. 1:**
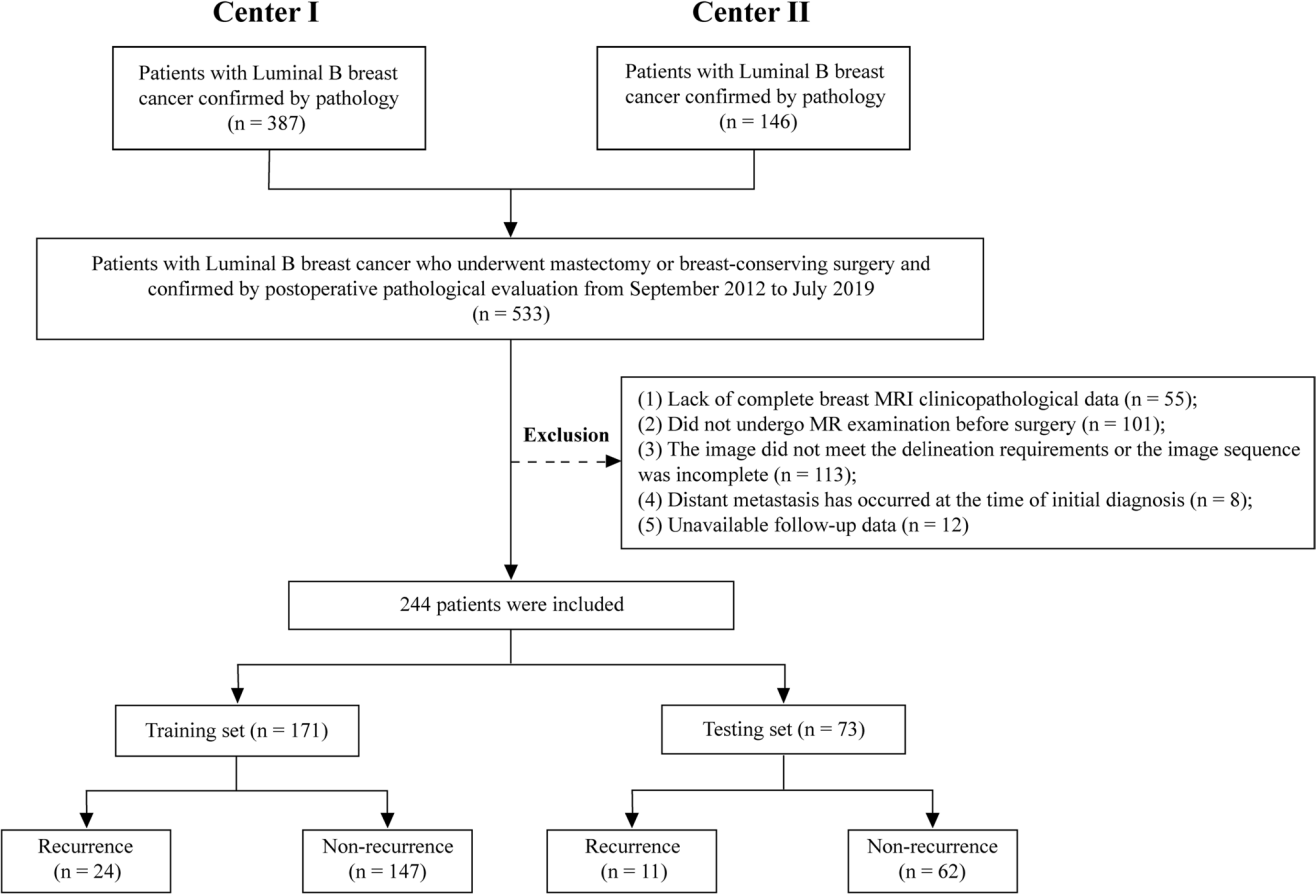
A flow chart of the patient recruitment process in this study

This study was conducted under the guidance of the TRIPOD Principles and the Declaration of Helsinki [16]. The Ethics Committees of two institutions approved the study, and the requirement for informed consent was waived because the study was retrospective. A total of 533 patients with luminal B breast cancer who underwent mastectomy or breast-conserving surgery between September 2012 and July 2019 were enrolled at both institutions. Ultimately, 244 patients were included in the study based on the inclusion and exclusion criteria outlined in Fig. 1.

Clinical information was obtained from medical records, including age, menopausal status, clinical stage, pathological stage, type of surgery, neoadjuvant chemotherapy (NAC), adjuvant chemoradiotherapy, endocrine therapy, timing and location of recurrences, and last follow-up.

Postoperative follow-up was conducted through telephone and outpatient visits. The patients were followed until 30 July 2021, and tumor recurrence was defined as locoregional recurrence (ipsilateral breast or chest wall and/or axilla, infraclavicular, or supraclavicular lymph nodes) or distant metastasis to other organs [17]. Recurrence-free survival (RFS) was defined as the period between surgery and the first recorded local or distant recurrence. A patient’s RFS was calculated as the time in days between the date of the initial operation and the last clinical follow-up visit if there was no recurrence.

### Histopathology

Histopathological data, including histological type, estrogen receptor (ER), progesterone receptor (PR), human epidermal growth factor receptor 2 (HER2), and Ki-67, were obtained from the histopathology report of the procedure. The expression levels of ER, PR, HER2, and Ki-67 were obtained from immunohistochemistry (IHC) analysis of the tumor tissue. The threshold values for ER and PR were set to 1%, and for Ki-67 to 14%. HER2 status was defined according to the American Society of Clinical Oncology (ASCO)/College of American Pathologists (CAP) guidelines [18]. Tumors with an IHC staining score of 0 or 1 + were classified as HER2 negative, while tumors with a score of 3 + were considered HER2 positive. For tumors with a score of 2 + , fluorescence in situ hybridization (FISH) was further performed to confirm HER2 status. Luminal B breast cancer was defined according to the 2013 St. Gallen Consensus Conference as ER-positive, HER2-negative, Ki-67 ≥ 14% patients, or ER-positive, HER2-positive [19].

### MRI Protocol and Radiologics Evaluation

All MRI examinations were conducted using a 3 T system (Verio, Siemens Healthcare, Erlangen, Germany) and a 16-channel dedicated breast coil at Center I and Center II.

The patient was positioned in a prone position with both breasts naturally suspended in a dedicated breast coil. The MRI protocol included a T2-weighted (T2WI) spin-echo series with fat suppression, axial diffusion-weighted imaging (DWI), and dynamic contrast-enhanced MRI (DCE-MRI). The DCE-MRI protocol comprised 1 pre- and 5 postcontrast axial image acquisitions using gadopentetate dimeglumine (Gd-DTPA; Beijing Beilu Pharmaceutical Co., Ltd., Beijing, China) injected into the median cubital vein at a dose of 0.1 mmol/kg, followed by 15 mL of normal saline at a rate of 2.0 mL/s. The apparent diffusion coefficient (ADC) map was generated by fitting the lowest and highest *b*-values (*b* = 0 mm^2^/s and *b* = 800 mm^2^/s) of the DWI map with a single exponential inline fit. Detailed MRI sequence parameters are shown in the Supplementary Tables 1–3.

All MRI data were transferred to a dedicated workstation for further analysis. The Imaging features were evaluated by two radiologists with 10 and 27 years of experience in breast imaging. Both radiologists were aware of the diagnosis of luminal B breast cancer but were blinded to the clinical and histopathological findings of the patients. The following imaging features are assessed: lesion morphology, lesion margin, time-intensity curve (TIC), maximum tumor diameter, and ADC mean. Lesion morphology (round/oval vs. irregular), lesion margin (circumscribed vs. not circumscribed), and TIC were evaluated on DCE-MRI by two radiologists in consensus according to the 2013 Breast Imaging Reporting and Data System (BI-RADS) MR imaging vocabulary standards proposed by the American College of Radiology. The maximum tumor diameter and ADC mean were measured on the DCE-MRI and ADC maps, respectively.

### Region-of-Interest Segmentation and Radiomics Feature Extraction

**Fig. 2:**
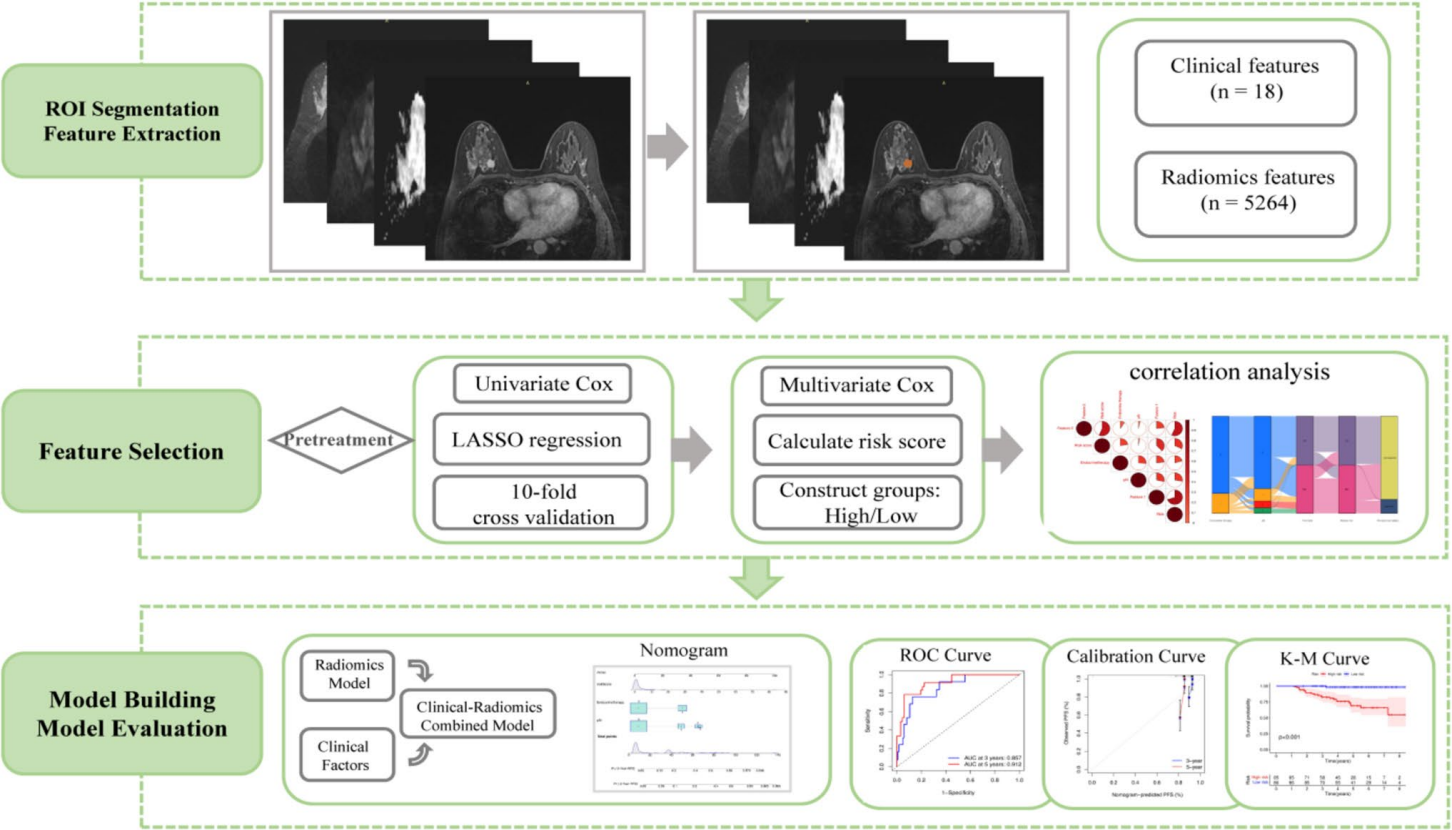
Workflow of model construction

All MRI images were anonymized and stored in Digital Imaging and Communications in Medicine (DICOM) format. The study flow is depicted in Fig. 2. Four series of images, including T2WI, DWI (*b* = 800 mm^2^/s), the DWI-derived ADC map, and the DCE-MRI arterial phase at the second scan, were exported from the picture archiving and communication system (PACS). A radiologist with 10 years of experience contoured each 3-dimensional tumor region of interest (ROI) using ITK-SNAP (version 3.80; http://www.itksnap.org/pmwiki/pmwiki.php) for each series of images, which was then reviewed by an experienced senior radiologist with 27 years of experience. The senior radiologist manually modified the lesion outline, if necessary, but did not perform the segmentation independently.

Image preprocessing and feature extraction were performed using the open-source Artificial Intelligence Kit platform (AK, version 3.2.2, GE Healthcare). We extracted a variety of features, including original feature shape, first-order features, gray-level co-occurrence matrix (GLCM), gray-level run length matrix (GLRLM), gray-level size zone matrix (GLSZM), neighboring gray-tone difference matrix (NGTDM), and gray-level dependence matrix (GLDM). We also filtered features Laplacian of Gaussian (LoG), wavelet, and local binary pattern (LBP) from 4 series of images, respectively.

### Feature Selection and Radiomics Signature Building

All features were preprocessed in three steps before feature filtering: (1) batch correction for multicenter data was conducted; (2) features with variance values close to zero in the radiomics data were identified and removed using the nearZeroVar function; and (3) the medianImpute and range methods of the preProcess function were used to process missing values and normalize data.

The following steps were then used for feature filtering: (1) the training set data were first analyzed based on univariate Cox risk regression, and the features with *P* < 0.001 were retained; (2) for the retained features, the least absolute shrinkage and selection operator (LASSO) method with tenfold cross-validation was performed to obtain the optimal data subset; (3) the multivariate Cox-regression analysis was used to filter out retained features as well as correlation coefficients; and (4) the risk score for each sample was then calculated based on the features and correlation coefficients. Subsequently, the interaction between the risk score and the RFS was determined for both the training and test sets. The receiver operating characteristic (ROC) curves were used to assess the performance of the risk score.

From the median risk score obtained from the training group, patients were divided into high- and low-risk groups. The Kaplan–Meier (KM) method was used to compare the survivability probability of patients in the high- and low-risk groups, and the log-rank test was used for comparison.

### Development of the Radiomics Nomogram

Univariate and multivariate Cox analyses were performed on the risk score and with clinicopathological features to identify features significantly associated with cancer recurrence at *P* < 0.05. The correlation between the relevant features and the risk of recurrence was subsequently evaluated.

The radiomics nomogram was developed by combining the recurrence-related features to calculate the nomogram risk score. The correlation between recurrence-related features, nomogram risk score, and patient status was also analyzed. The ROC curves were used to assess the performance of the nomogram in stratifying the recurrence of luminal B cancer, and area under the curve (AUC) values were calculated. Calibration curves were then employed to determine the robustness of the model in both the training and testing sets.

### Statistical Analysis

All statistical analyses were performed using R software (version 4.0.5, https://www.r-project.org). The clinicopathological features of patients in the training and testing sets were compared by the Mann–Whitney *U* test or *t*-test for continuous variables and the Chi-squared or Fisher exact test for categorical variables. A two-tailed *P* < 0.05 was considered statistically significant.

## Results

### Patient Characteristics

A total of 244 patients were divided randomly into a training set (*n* = 171, 51.5 ± 12.5 years old) and a testing set (*n* = 73, 51.7 ± 11.3 years old) in a ratio of 7:3. Baseline clinical characteristics of patients in the training and testing sets were comparable, and no significant difference was observed (*P* > 0.05, Table 1). At the last follow-up, 35 patients had experienced disease recurrence, with 24 in the training set and 11 in the testing set. Of these, 17 patients only had local recurrence, and 18 had distant metastases. The estimated median follow-up time was 4.58 years.

**Table 1:** Clinical and pathological characteristics of luminal B patients in the training and testing sets

**Characteristics**	**Training set (*n*** **= 171)**	**Testing set (*n*** **= 73)**	***P*** **value**	*P *value*
**Age(years ± SD)**	51.5 ± 12.5	51.7 ± 11.3	0.899	0.654
**Tumor size(mm)**	23.9 ± 13.2	25.2 ± 13.2	0.504	0.181
**Menopausal status (%)**				
**Premenopausal**	74 (43.3%)	37 (50.7%)	0.287	
**Postmenopausal**	97 (56.7%)	36 (49.3%)		
**Histological type (%)**				
** IDC**	154 (90.1%)	65 (89.0%)	0.889	
** ILC**	7 (4.1%)	4 (5.5%)		
** Others**	10 (5.8%)	4 (5.5%)		
**TIC pattern (%)**				
** Type I**	29 (17.0%)	9 (12.3%)	0.636	
** Type II**	56 (32.7%)	24 (32.9%)		
** Type III**	86 (50.3%)	40 (54.8%)		
**Mass shape (%)**				
** Round/oval**	40 (23.4%)	18 (24.7%)	0.832	
** Irregular**	131 (76.6%)	55 (75.3%)		
**Margin (%)**				
** Circumscribed**	18 (10.5%)	7 (9.6%)	0.825	
** Not circumscribed**	153 (89.5%)	66 (90.4%)		
**BI-RADS (%)**				
** 2**	3 (1.8%)	0 (0.0%)	0.565	
** 3**	8 (4.7%)	1 (1.4%)		
** 4**	53 (31.0%)	24 (32.9%)		
** 5**	102 (59.6%)	46 (63.0%)		
** 6**	5 (2.9%)	2 (2.7%)		
**ADC mean**	635.1 ± 132.4	609.5 ± 153.2	0.189	0.253
**Clinical stage (%)**				
** 1**	77 (45.0%)	26 (35.6%)	0.378	
** 2**	72 (42.1%)	37 (50.7%)		
** 3**	22 (12.9%)	10 (13.7%)		
**pT (%)**				
** 1**	88 (51.5%)	31 (42.5%)	0.185	
** 2**	68 (39.8%)	39 (53.4%)		
** 3**	13 (7.6%)	3 (4.1%)		
** 4**	2 (1.2%)	0 (0.0%)		
**pN (%)**				
** 0**	129 (75.4%)	53 (72.6%)	0.256	
** 1**	21 (12.3%)	10 (13.7%)		
** 2**	14 (8.2%)	3 (4.1%)		
** 3**	7 (4.1%)	7 (9.6%)		
**PR(%)**				
** Negative**	16 (9.4%)	7 (9.6%)	0.955	
** Positive**	155 (90.6%)	66 (90.4%)		
**HER2 (%)**				
** Negative**	114 (66.7%)	41 (56.2%)	0.119	
** Positive**	57 (33.3%)	32 (43.8%)		
**Type of surgery (%)**				
** Mastectomy**	119 (69.6%)	53 (72.6%)	0.637	
** BCS**	52 (30.4%)	20 (27.4%)		
**NAC (%)**				
** No**	155 (90.6%)	65 (89.0%)	0.700	
** Yes**	16 (9.4%)	8 (11.0%)		
**Endocrine therapy (%)**				
** No**	134 (78.4%)	60 (82.2%)	0.497	
** Yes**	37 (21.6%)	13 (17.8%)		
**Adjuvant chemoradiotherapy (%)**				
** No**	29 (17.0%)	12 (16.4%)	0.921	
** Yes**	142 (83.0%)	61 (83.6%)		

*IDC* invasive ductal carcinoma, *ILC* invasive lobular carcinoma, *TIC* time-intensity curve, *ADC* apparent diffusion coefficient, *PR* progesterone receptor, *HER2* human epidermal growth factor receptor, *BCS* breast-conserving surgery, *NAC* neoadjuvant chemotherapy

^*^In the case that the *t*-test is not satisfied, the Mann–Whitney *U* test is used

### Radiomics Feature Selection and Signature Building

A total of 5264 radiomics features were extracted from each patient in this study, including 1316 features each for DWI, ADC, and DCE-MRI arterial phase at the second scan and T2WI sequences. After feature selection using univariate Cox risk regression analysis, 230 features were identified. Further feature reduction was carried out using LASSO regression with tenfold cross-validation, resulting in two remaining features. After stepwise multifactor Cox risk regression, two features from DCE-MRI were selected. The risk score in each experimental sample was determined using the characteristics and correlation coefficients, and the calculation formula was as follows:

Riskscore = exp(lbp_3D_m1_glrlm_LongRunLowGrayLevelEmphasis * 3.447871 + wavelet_HLH_glszm_SizeZoneNonUniformityNormalized * 4.966189 + (− 2.783055)).

**Fig. 3:**
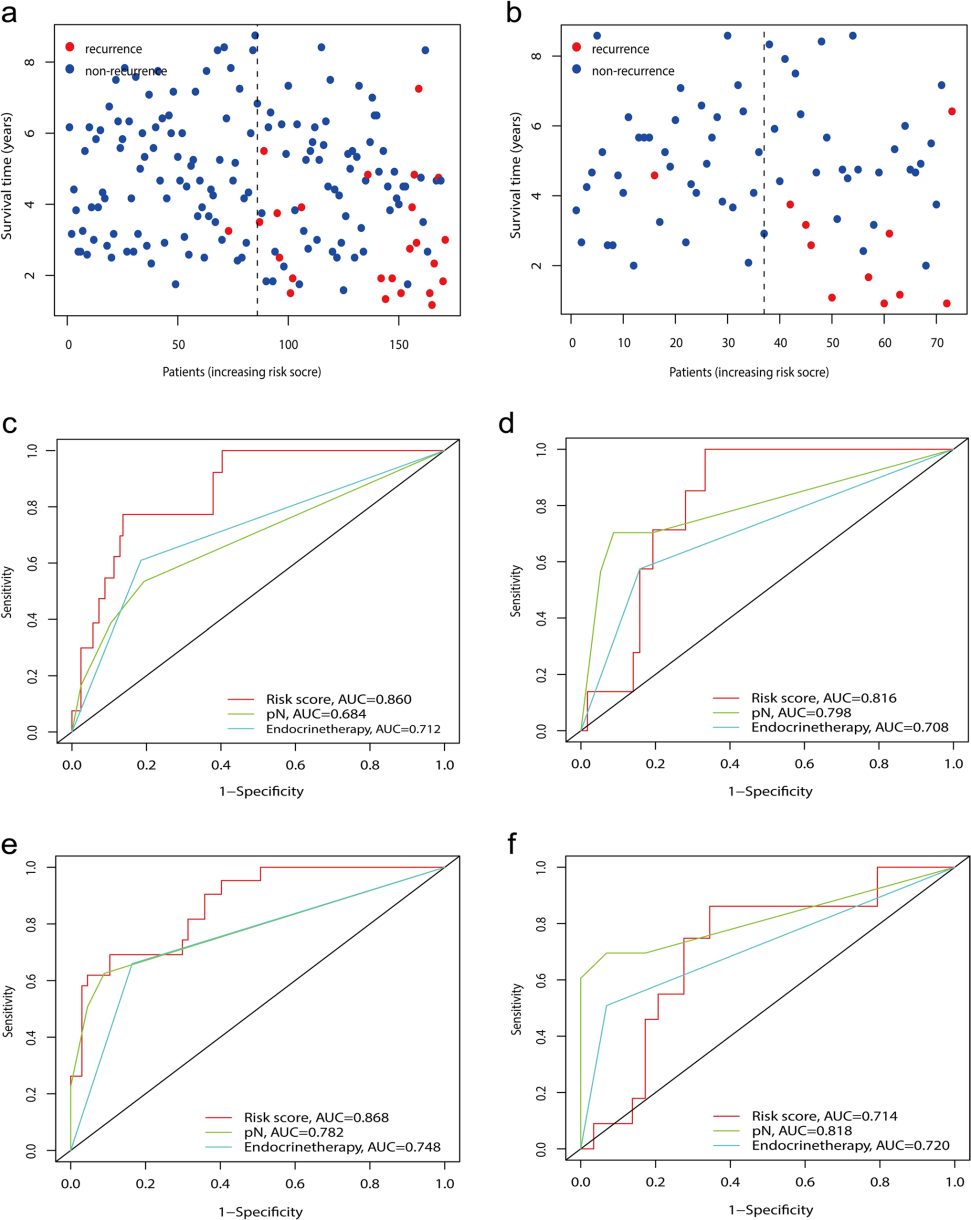
Performance of the model and clinical features. **a**, **b** The relation of the risk score and recurrence-free survival (RFS) for non-recurrence and recurrence patients in the training and testing sets, respectively, and **c**–**f** the ROC curves of the risk score and clinical factors for 3 and 5 years in the training and testing sets, respectively

The relationship between risk score and RFS for non-recurrence and recurrence patients is shown in Fig. 3. The risk score demonstrated good performance in stratifying luminal B recurrence, with AUC values of 0.860 and 0.868 in the training set and 0.816 and 0.714 in the testing set for 3 and 5 years, respectively (Fig. 3c–f).

**Fig. 4:**
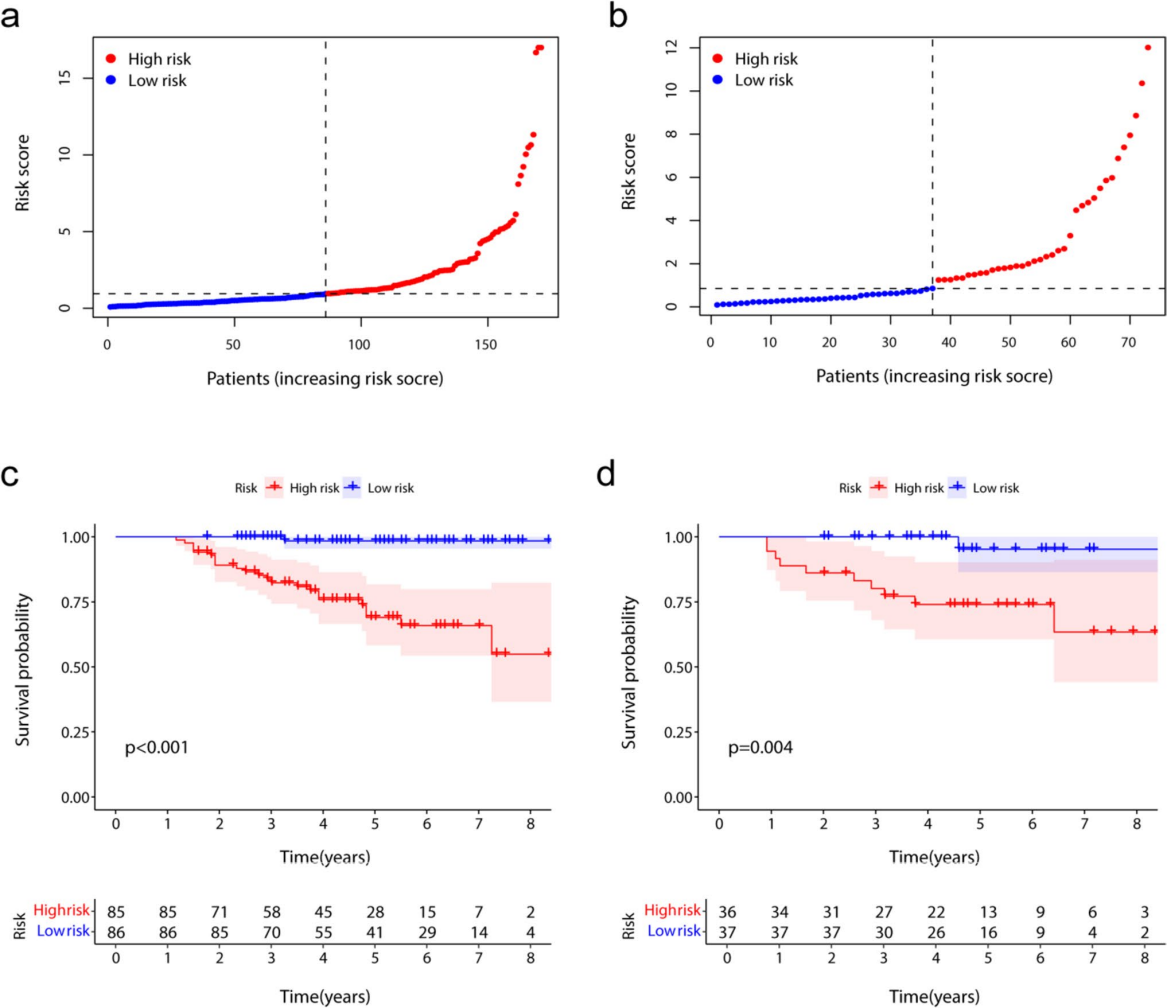
The risk grouping of patients and the survival analysis of patients with two risk groups. **a**, **b** The plots show the low- and high-risk groups divided from the median value of the risk score in the training and testing sets, respectively. **c**, **d** The plots show the Kaplan–Meier survival analysis of two risk groups in the training and testing sets

Using a risk score cut-off of 0.946, patients were categorized into high-risk (risk score > 0.946) and low-risk (risk score ≤ 0.946) groups. The KM survival curves showed significant differences in RFS between low- and high-risk patients (Fig. 4).

### Development and Performance of the Radiomics Nomogram

**Fig. 5:**
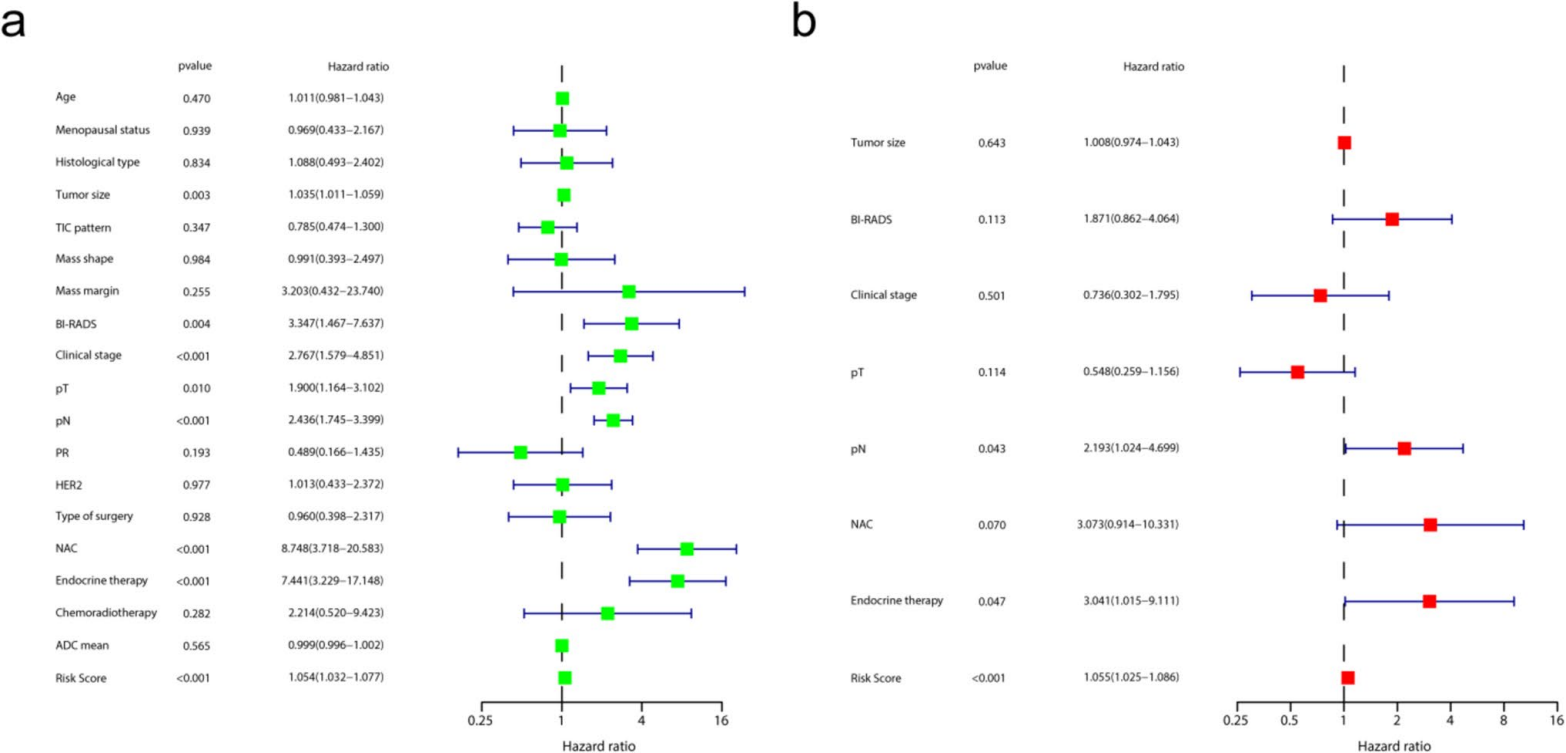
Univariate and multivariable Cox regression analyses of predictors of RFS in the training set. Hazard ratios and 95% confidence intervals (CIs) were used to show the correlation between the recurrence risk and factors including clinicopathological features and risk score. TIC, time-intensity curve; PR, progesterone receptor; HER2, human epidermal growth factor receptor 2; NAC, neoadjuvant chemotherapy; ADC, apparent diffusion coefficient

**Fig. 6:**
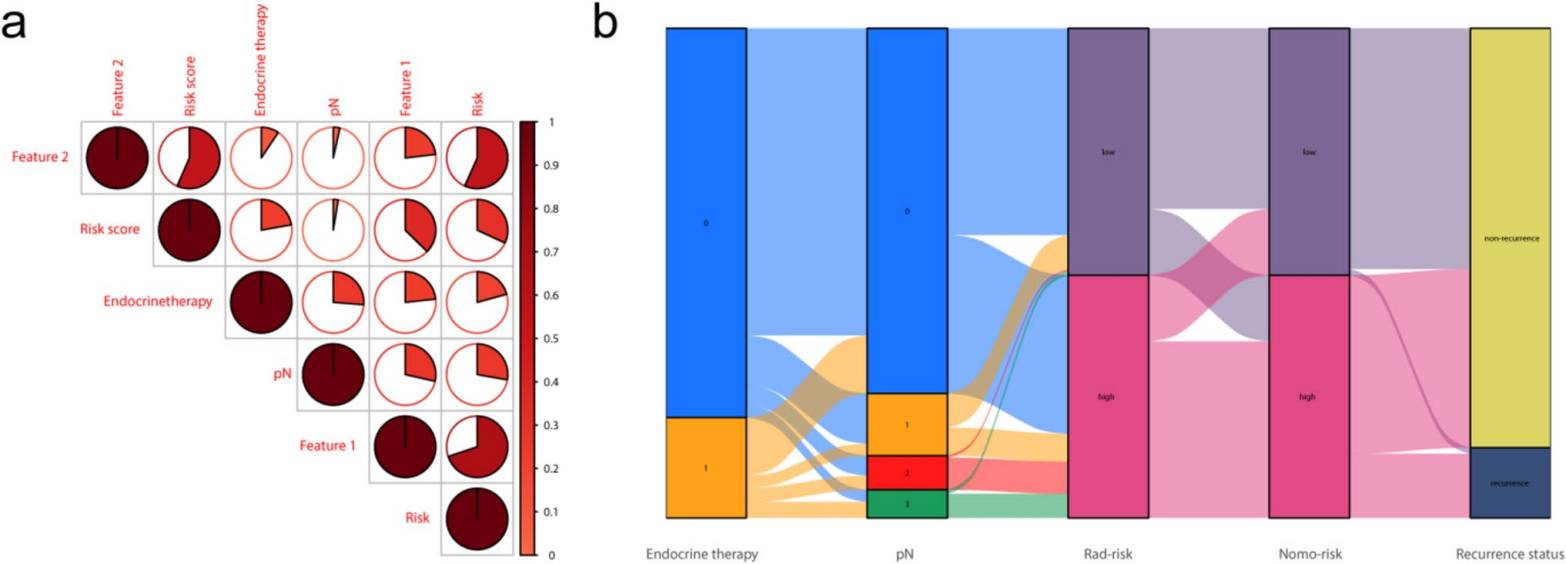
Correlation analysis. **a** The relation of the radiomics signatures and the clinical features screened. **b** The Sankey diagram showed the correlation of endocrine therapy, pN, rad-risk, nomo-risk, and recurrence status of patients. Feature1 = lbp_3D_m1_glrlm_LongRunLowGrayLevelEmphasis; Feature2 = wavelet_HLH_glszm_SizeZoneNonUniformityNormalized

The risk score and clinicopathological characteristics were subjected to univariate Cox and multivariate Cox analyses, and pN, endocrine therapy, and risk score were identified as independent stratification measures of luminal B breast cancer recurrence (Fig. 5). The correlation between selected radiomics features, clinicopathological variables, risk score, and recurrence risk is shown in Fig. 6a.

**Fig. 7:**
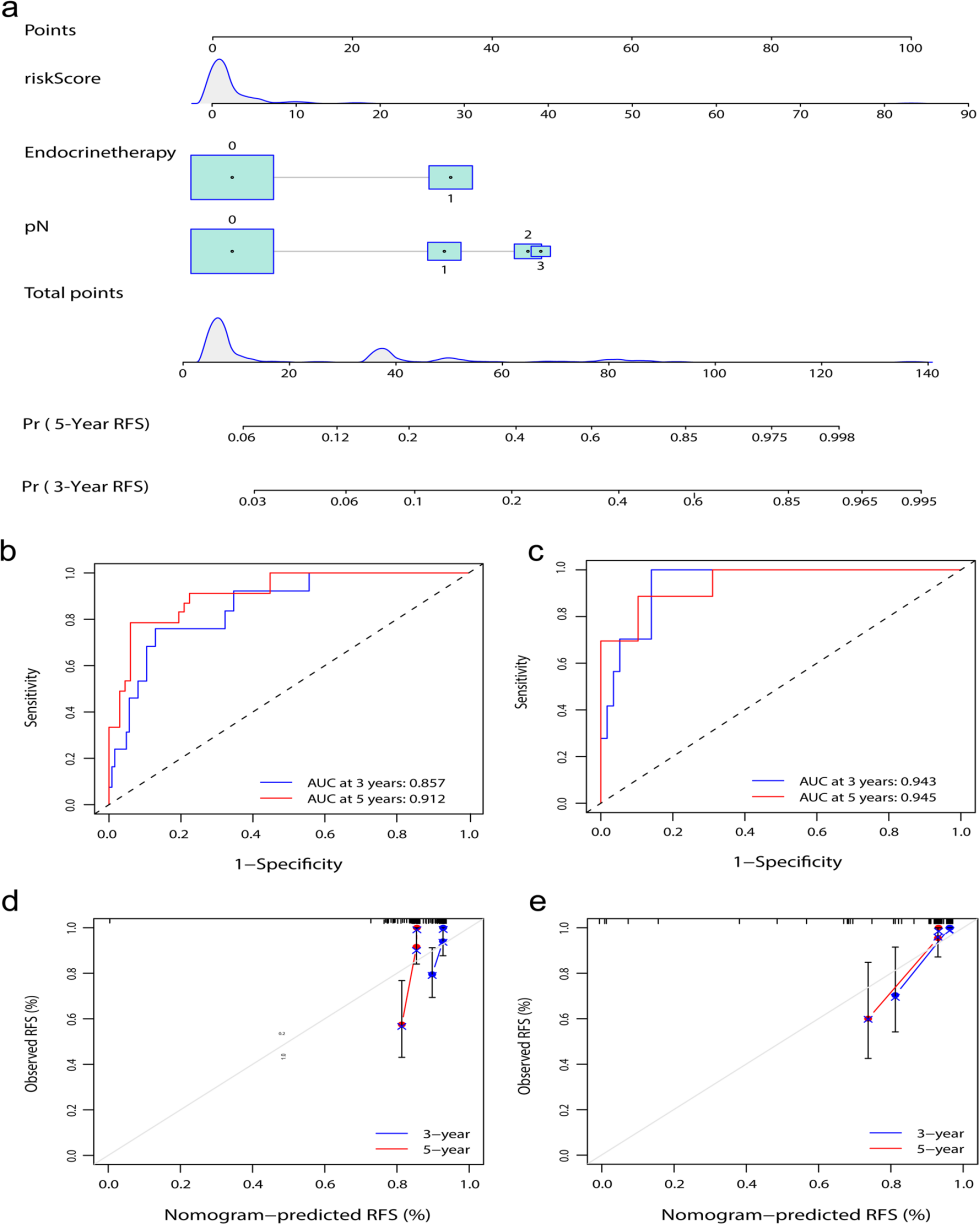
Construction and performance of combined models. **a** Development of radiomics nomogram. The nomogram was constructed by combining the risk score, pN, and endocrine therapy. **b**, **c** The plots display the ROC curves of the combined model for 3 years and 5 years in the training and testing sets, respectively. **d**, **e** The plots show the calibration curves for 3 years and 5 years in the training and testing sets, respectively

A nomogram was constructed based on the combined model using the risk score, pN, and endocrine therapy. The patients with three independent risk factors (risk score, pN, and endocrine therapy), nomogram risk score, and recurrence status were also shown in the Sankey diagram (Fig. 6b). The nomogram was drawn based on the combined model (Fig. 7a). The combined model showed good discriminative power, with AUC values of 0.857 and 0.912 in the training set and 0.943 and 0.945 in the testing set over 3 and 5 years, respectively (Fig. 7b, c). The calibration curve of the combined model indicated good agreement between the predicted and measured values (Fig. 7d, e).

**Fig. 8:**
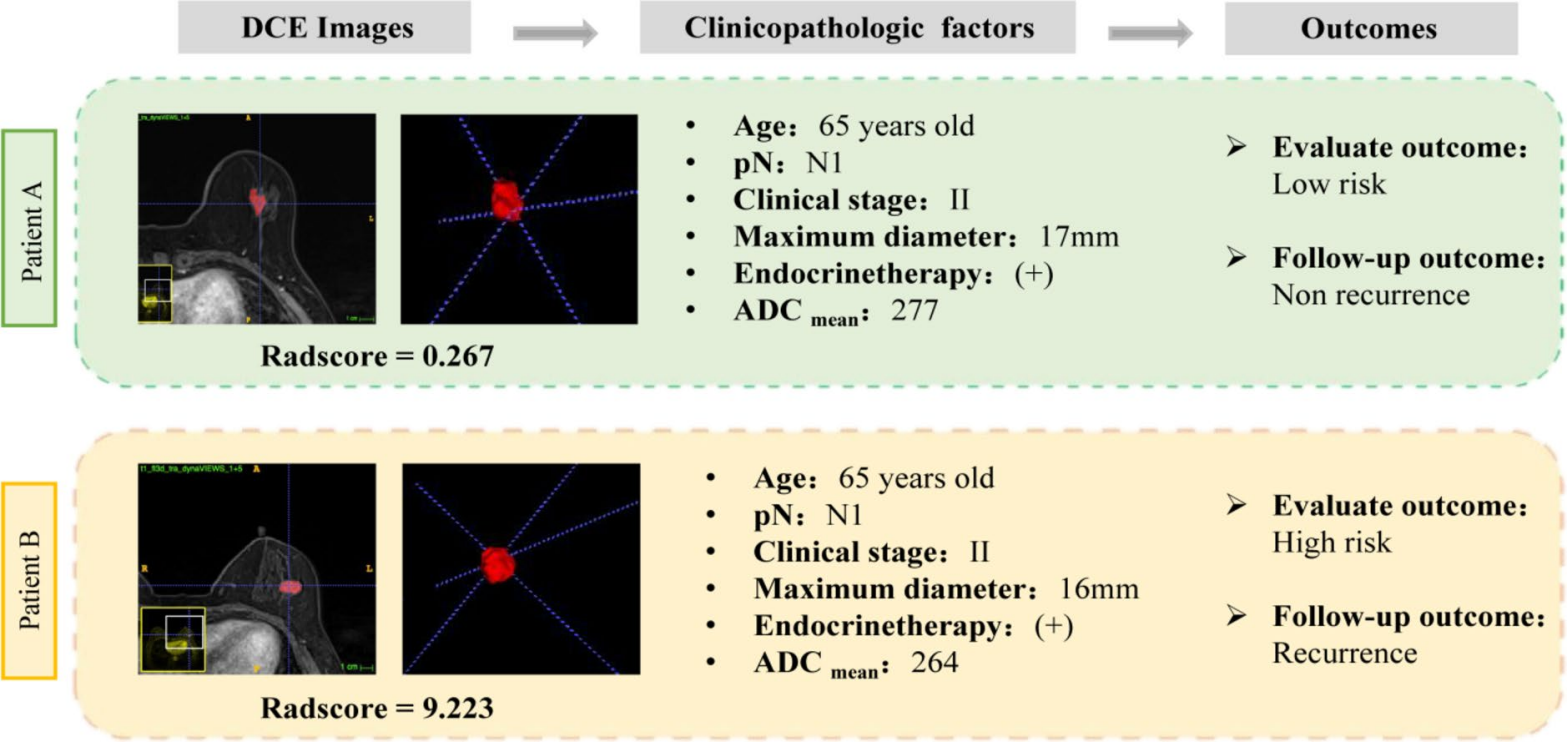
Two typical cases demonstrated the clinical transformation of radiomics models. Two presented cases of luminal B patients who had similar clinicopathological features were assigned to high-risk and low-risk groups according to the risk score, respectively

Additionally, two representative cases of luminal B breast cancer patients with similar clinicopathological factors were presented, and the radiomics model correctly distinguished the high and low recurrence risk of patients (Fig. 8).

## Discussion

This study developed a postoperative recurrence stratification model for luminal B breast cancer based on radiomics features extracted from four MRI sequences. We screened 2 radiomics features from DCE-MRI that were found to have independent values for stratifying luminal B recurrence in univariate and multifactorial Cox analyses, which was consistent with previous studies reported [20, 21]. Furthermore, the risk score and the combined model had a better ability to stratify the risk of recurrence compared to traditional clinicopathological factors, contributing to individualized clinical treatment decisions.

Previous studies have also used radiomics to assess the recurrence of hormone receptor-positive breast cancer. For example, the model developed by Jacobs et al. combining clinicopathological, imaging, and multisequence radiomics features was discovered to help develop a comprehensive understanding of hormone receptor-positive tumors and their surrounding tissues, thus improving recurrence risk assessment [22]. Building on the study by Jacobs et al., Romeo et al. found that combining MRI radiomics and machine learning helps to noninvasively predict the risk of recurrence in patients with hormone receptor-positive breast cancer [23]. However, these studies were focused on the overall hormone receptor-positive breast cancer patients rather than luminal A or luminal B subtypes, which differ greatly in aggressiveness and treatment response, and thus modeling for individual subtypes is more conducive to guiding precise clinical judgments. Xiong et al. used radiomics features of preoperative ultrasound to predict RFS in patients with luminal B breast cancer, and ultimately, the model significantly differentiated high- and low-risk recurrence populations [15]. The study, however, was based on ultrasound radiomics, whereas MRI is a more precise tool for preoperative staging and prognostic assessment of breast cancer. Accordingly, this study developed a predictive model based on MRI radiomics for the recurrence of luminal B breast cancer.

In this study, DCE-MRI was used to develop a predictive model for the recurrence of luminal B breast cancer. We found that DCE-MRI can reflect lesion morphology, boundary, margin, and blood supply and distribution characteristics more accurately than standard MRI and thus better reflect lesion characteristics. The findings herein are similar to those of Kamiya et al., who explored the relationship between texture features and RFS by preoperative T2WI and DCE-MRI in TNBC patients [24]. Their study ultimately concluded that only DCE-MRI features were independently associated with RFS.

Certain reports in the literature differ from our findings, which indicated that T2WI captured tumor heterogeneity equally well or better than DCE-MRI [8, 25]. According to Eun et al., there was no statistical difference between T2WI and DCE-MRI when examined against each other [17]. This may be the result of utilizing different scanners and sequence parameters or differences in the study population and the purpose of their study.

Regardless, DCE-MRI can reflect blood supply to the tumor through a variety of dynamic enhancement patterns, which may improve the accuracy of identifying tumor margins [26, 27]. In addition, other sequences are rarely used in radiomics workflows due to their lower resolution [28]. Therefore, DCE-MRI is still considered the best tool for forecasting the outlook of breast cancer recurrence using radiomics workflows.

The model developed in this study achieved a relatively good AUC, which is consistent with the performance of the DCE-MRI model developed in the study by Ma et al. to predict the risk of TNBC recurrence [29]. Although many studies have concluded that multisequence MRI radiomics models can reveal tumor heterogeneity from multiple perspectives, our model showed similar performance to the multisequence model developed in the study by Rabinovici-Cohen et al. for predicting the recurrence of hormone receptor-positive breast cancer [30–32].

Moreover, the use of single-sequence models may be beneficial because radiomics studies have not yet developed a standardized MRI scanning protocol, and reconstruction algorithms and scan parameters can vary widely among MR providers and institutions. These variables affect texture features and the robustness of radiomics models. If the performance and robustness of single-sequence models can be confirmed by larger studies, they will provide greater benefits and potential for the clinical application and effectiveness of radiomics models.

In this study, two texture features from GLSZM and GLRLM were found to be related to the recurrence of luminal B breast cancer. Both features were derived from filtered features and can be difficult to interpret intuitively in clinical practice. Under radiomics guidance, these high-dimensional images can be assessed with sensitivity and specificity, greatly contributing to the monitoring of neoadjuvant therapy, lymph node metastasis, and gene expression [33–35].

### Limitations

The retrospective nature of this study may have led to selection bias, which may affect the generalizability of the results. In addition, the small sample size may limit the power of the study, and future studies with larger sample sizes are needed to confirm the results. Although data from both institutions were included in this study, the equipment parameters were similar, and future studies should include multiple MRI protocols and external validation to ensure the model’s compatibility with different MRI scanners.

## Conclusion

In conclusion, this study developed a predictive model for the recurrence of luminal B breast cancer based on DCE-MRI radiomics features. Due to the importance of proper diagnosis and evaluation in treating luminal B breast cancer, the algorithm proposed herein may offer a high degree of clinical utility in guiding personalized treatment.

### Supplementary Information

Below is the link to the electronic supplementary material.

Supplementary file1 (DOCX 16 KB)

## Data Availability

The data that support the findings of this study have been originated by the First Affiliated Hospital of Zhejiang Chinese Medical University and Hangzhou First People’s Hospital, but restrictions apply to the availability of these data, which were used under license for the current study and so are not publicly available. Data are, however, available from the corresponding author on reasonable request.
