# Correction: A Multiparametric MRI-based Radiomics Model for Stratifying Postoperative Recurrence in Luminal B Breast Cancer

**DOI:** 10.1007/s10278-024-01177-9

**Published:** 2024-06-28

**Authors:** Kepei Xu, Meiqi Hua, Ting Mai, Xiaojing Ren, Xiaozheng Fang, Chunjie Wang, Min Ge, Hua Qian, Maosheng Xu, Ruixin Zhang

**Affiliations:** 1 Department of Radiology, The First Affiliated Hospital of Zhejiang Chinese Medical University (Zhejiang Provincial Hospital of Chinese Medicine), 54 Youdian Road, Hangzhou, China; 2 The First School of Clinical Medicine, Zhejiang Chinese Medical University, Hangzhou, China; 3 Department of Radiology, Hangzhou First People’s Hospital, Zhejiang Province Hangzhou, China

**Correction to: Journal of Imaging Informatics in Medicine** 10.1007/s10278-023-00923-9

There is a mistake in the affiliation listing for author Ruixin Zhang. The correct affiliation listing is:

- The First Affiliated Hospital of Zhejiang Chinese Medical University (Zhejiang Provincial Hospital of Chinese Medicine)

