# Supplementary material for: A Multiparametric MRI-based Radiomics Model for Stratifying Postoperative Recurrence in Luminal B Breast Cancer
Source: J Imaging Inform Med. 2024 Feb 29;37(4):1475–87. doi: 10.1007/s10278-023-00923-9 (PMC11300413; doi:10.1007/s10278-023-00923-9)
Supplement: Supplementary file 1 — Supplementary file1 (DOCX 16 KB) [file 10278_2023_923_MOESM1_ESM.docx]

**A Multiparametric MRI-based Radiomic Model for Stratifying** [**Postoperative**](javascript:;) **Recurrence in Luminal B Breast Cancer**

Kepei Xu, Meiqi Hua, Ting Mai, Xiaojing Ren, Xiaozheng Fang, Chunjie Wang, Min Ge, Hua Qian, Maosheng Xu*, Ruixin Zhang*

***: Corresponding Author Info:**

Maosheng Xu, Department of Radiology, The First Affiliated Hospital of Zhejiang Chinese Medical University (Zhejiang Hospital of Traditional Chinese Medicine), 54 Youdian Road, Hangzhou, China; E-mail: xums166@zcmu.edu.cn

Ruixin Zhang, Department of Radiology, The First Affiliated Hospital of Zhejiang Chinese Medical University (Zhejiang Hospital of Traditional Chinese Medicine), 54 Youdian Road, Hangzhou, China; E-mail: [ruixinr@zcmu.edu.cn](mailto:ruixinr@163.com)

## **SUPPLEMENT**

*Supplemental Table 1. DCE-MRI scan parameters*

| Scanner | Scanner I (3T) | Scanner II (3T) |
| --- | --- | --- |
| Sequence | 3D fast low-angle shot  (FLASH) | 3D fast low-angle shot  (FLASH) |
| Orientation | Axial | Axial |
| Fat suppression | Quick Fat Sat | Quick Fat Sat |
| Repetition time (msec) | 4.51 | 4.51 |
| Echo time (msec) | 1.61 | 1.61 |
| Field of view (mm^2^) | 340×340 | 340×340 |
| Matrix | 448×448 | 448×300 |
| Number of slices | 144 | 160 |

*Supplemental Table 2. DWI scan parameters*

| Scanner | Scanner I (3T) | Scanner II (3T) |
| --- | --- | --- |
| Sequence | Single-shot spin-echo EPI with bipolar diffusion pulses | Single-shot spin-echo EPI with bipolar diffusion pulses |
| Orientation | Axial | Axial |
| Diffusion directions | Three-direction trace | Three-direction trace |
| Orientation | Axial | Axial |
| *b value (sec/mm2)* | 50, 800 | 50, 800 |
| Fat suppression | Spectral Attenuated Inversion Recovery | Spectral Attenuated Inversion Recovery |
| Repetition time (msec) | 8400 | 10800 |
| Echo time (msec) | 84 | 85 |
| Field of view (mm) | 360×167 | 370×182 |
| Matrix | 220×110 | 220×220 |
| Number of slices | 24 | 24 |

EPI=echo-planar imaging.

*Supplemental Table 3. T2WI scan parameters*

| Scanner | Scanner I (3T) | Scanner II (3T) |
| --- | --- | --- |
| Sequence | T2-weighted fat-suppressed spin-echo  (FSE) | T2-weighted fat-suppressed spin-echo  (FSE) |
| Orientation | Axial | Axial |
| Repetition time (msec) | 4000 | 4000 |
| Echo time (msec) | 70 | 70 |
| Field of view (mm^2^) FOV | 360×360 | 340×340 |
| Matrix | 448×448 | 448×448 |
| Number of slices | 34 | 34 |
| Slice thickness (mm) | 4.00 | 4.00 |

Gd-DTPA: gadopentetate dimeglumine
